# Monocarboxylate transporter 1 blockade with AZD3965 inhibits lipid biosynthesis and increases tumour immune cell infiltration

**DOI:** 10.1038/s41416-019-0717-x

**Published:** 2020-01-15

**Authors:** Mounia Beloueche-Babari, Teresa Casals Galobart, Teresa Delgado-Goni, Slawomir Wantuch, Harold G. Parkes, Debbie Tandy, James A. Harker, Martin O. Leach

**Affiliations:** 1 Cancer Research UK Cancer Imaging Centre, The Institute of Cancer Research and The Royal Marsden NHS Foundation Trust, London, SW7 3RP UK; 2 Cancer Research UK Cancer Therapeutics Unit, The Institute of Cancer Research and The Royal Marsden NHS Foundation Trust, SW7 3RP, London, UK; 3 National Heart and Lung Institute, Imperial College London, SW7 2AZ, London, UK

**Keywords:** Targeted therapies, Cancer metabolism

## Abstract

**Background:**

Monocarboxylate transporter 1 (MCT1) is a regulator of cell metabolism and a therapeutic target for cancer treatment. Understanding the changes in tumour function accompanying MCT1 inhibition will better characterise the anti-tumour effects of MCT1 inhibitors, potentially enabling the identification of pharmacodynamic biomarkers for the clinical development of these agents.

**Methods:**

We assessed the impact of the MCT1 inhibitor AZD3965 on tumour metabolism and immune cell infiltration as key determinants of tumour biological function in the MCT1-dependent Raji B cell lymphoma model.

**Results:**

Treatment of Raji xenograft-bearing severe combined immunodeficiency mice with AZD3965 led to inhibition of tumour growth paralleled with a decrease in tumour choline, as detected by non-invasive in vivo proton nuclear magnetic resonance spectroscopy. This effect was attributed to inhibition of phosphocholine de novo synthesis following decreased choline kinase α protein and messenger RNA expression that correlated with the AZD3965-induced build-up in intracellular lactate. These changes were concomitant with increased tumour immune cell infiltration involving dendritic and natural killer cells.

**Conclusions:**

Our data provide new insights into the metabolic and cellular changes that occur in the tumour microenvironment following MCT1 blockade, which may contribute to the anti-tumour activity of AZD3965 and could have potential as pharmacodynamic biomarkers of MCT1 inhibition.

## Background

Metabolic transformation describes the ability of cancer cells to upregulate the metabolism of many key nutrients such as glucose, glutamine and lipids to support the cells’ increased biomass-building requirements and need to maintain viability and function in the challenging, nutrient- and oxygen-limited tumour microenvironment.^1^ This metabolic reprogramming is enabled through oncogenic signalling pathways (e.g. phosphatidylinositol-3-kinase/AKT/mammalian target of rapamycin pathway) and/or changes in the tumour microenvironment (e.g. hypoxia) that are intricately linked to the regulation of metabolic gene expression, in addition to mutation/overexpression of metabolic enzymes themselves.^1,2^

One example of the many effectors of metabolic re-wiring in cancer is the monocarboxylate transporter (MCT) family (including MCT1, MCT2, MCT3 and MCT4), which are transmembrane proteins that mediate the bi-directional transport of lactate (as well as other substrates such as pyruvate, short-chain fatty acids and ketones) in and out of cells.^3^ MCTs allow the removal of excess lactate produced from increased glycolytic activity, which occurs even under aerobic conditions, thus preventing intracellular acidification. MCTs also mediate the uptake of lactate into cells, which can be used as a fuel source and oxidised to produce energy in a variety of cell types, including cancer, stromal and immune cells.^4,5^

The key role that MCTs play in regulating lactate homeostasis in tumours together with the upregulation of MCT1 and MCT4 expression observed in many cancers has led to much interest in targeting these proteins for cancer treatment.^4,6–8^ To date, several inhibitors of MCT1 have been reported that exert promising activity in experimental cancer models, including SR13800^9^ and AZD3965,^10^ and progress is being made towards the development of MCT4-targeted agents (AstraZeneca).

The MCT1 inhibitor AZD3965 is in early phase clinical trials (www.clinicaltrials.gov) with phase I expansion cohort enrichment for Burkitt lymphoma and diffuse large B cell lymphoma cases, given the promising pre-clinical results obtained with the drug in this setting.^11–13^ Gaining an insight into the consequences of MCT1 inhibition on metabolism and tumour function as a whole is therefore necessary to (a) improve our understanding of the processes relevant to the drug’s mechanism of action and (b) enable the discovery of pharmacodynamic (PD) biomarkers of target modulation.

In this regard, we have previously shown that blockade of MCT1 with AZD3965 has profound effects on human cancer cell metabolism that extend beyond intracellular lactate (lactate_i_) build-up. Indeed, in MCT4− cells (MCT4 expression being predictive of resistance to MCT1 inhibition^10,13^) we observed effects on mitochondrial activity, shown by increased oxidative metabolism and improved bioenergetics as well as decreased phosphocholine (PCho) levels, implying effects on phospholipid metabolism and alterations in several other metabolic intermediates.^13^

In addition to re-wiring global metabolic activity in cancer cells, MCT1 blockade is also expected to impact on the tumour microenvironment not least because lactate is a mediator of several processes within it, including angiogenesis, metabolic symbiosis between cancer and stromal cells, and immune suppression.^1,14,15^

Thus, in this study we sought to characterise the effect of MCT1 inhibition on tumour choline metabolism in vivo using non-invasive proton nuclear magnetic resonance (^1^H NMR) spectroscopy (MRS) and evaluate the accompanying changes in the tumour microenvironment.

We show that treatment with AZD3965 in a MCT4− tumour xenograft model blocks choline phospholipid synthesis in vivo as detected non-invasively with single voxel ^1^H MRS, and concomitantly increases immune cell infiltration in the tumour.

## Methods

### Cell lines and cell culture

The human cell lines Raji (B cell lymphoma) and Hut78 (T cell lymphoma) were purchased from ATCC. HT29 human colon carcinoma cells were originally obtained from ATCC and authenticated in our laboratory by STR profiling on 13 March 2017. The cell lines were grown in either RPMI (Roswell Park Memorial Institute) (Raji and Hut78) or DMEM (Dulbecco’s modified Eagle’s medium) (HT29) as previously described.^16^ The Raji cell line was chosen as an AZD3965-sensitive cell line (MCT4−), also representative of the phase I trial expansion cohort, while HT29 and Hut78 cell lines were chosen to provide a range of baseline expression of MCT4 (Hut78 was MCT4+ and HT29 was MCT4+++, as previously shown^13^). Cell culture reagents were purchased from Gibco and AZD3965 was kindly provided by AstraZeneca, UK. Cells were exposed to either dimethyl sulfoxide (DMSO) (0.01%) or AZD3965 in 0.01% DMSO for the indicated duration and at the indicated concentrations in fresh media.

### Western blotting and qRT-PCR

Western blotting and quantitative real-time PCR (qRT-PCR) for protein and gene expression analyses, respectively, were performed using standard conditions as previously described.^17^ Details of the primary and secondary antibodies as well as primers used are provided in Supplementary Materials and methods.

### Tumour xenograft model

Raji xenograft tumours were established by subcutaneous injection of Raji cells into the flank of female severe combined immunodeficiency (SCID) (CB17/Icr-*Prkdc*^*scid*^) mice (6–8 weeks old, ca. 20 g in weight) as previously described^13^ maintained in pathogen-free conditions within the Biological Services Unit laboratories, ICR. Tumours were measured as previously described^16^ and once they reached 300–350 mm^3^ in volume (after ~4 weeks), mice were randomised into two groups; one group was dosed with vehicle (85% saline, 10% DMSO and 5% Tween (*n* = 8)) and the other with 50 mg/kg AZD3965 dissolved in vehicle (*n* = 9) administered orally twice daily over 5 days. This schedule was based on a previously published report^10^ and shown in our laboratory to cause tumour lactate build-up consistent with MCT1 blockade.^13^ No adverse events were associated with the procedures carried out in the experiment.

All experimental protocols were monitored and approved by the ICR Animal Welfare and Ethical Review Body Animal Welfare and Ethical Review Body, in accordance with UK Home Office regulations under the Animals (Scientific Procedures) Act 1986 and UK National Cancer Research Institute (NCRI) Guidelines for the Welfare and Use of Animals in Cancer Research.^18^

### In vivo ^1^H MRS of tumours

**Fig. 1:**
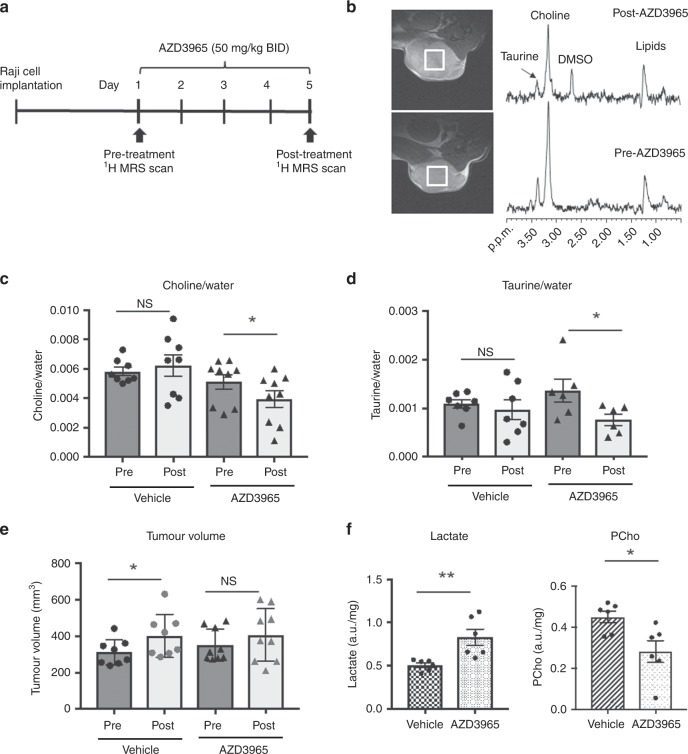
AZD3965 treatment decreases in vivo choline/water in Raji human lymphoma xenografts detected non-invasively with ^1^H MRS. **a** Schematic representation of the AZD3965 treatment and ^1^H MRS scanning schedule. **b** Representative T2-weighted anatomical images with the ^1^H MRS localisation volume (square, left) and ^1^H MRS spectra (right) acquired from a Raji tumour-bearing mouse pre- and post treatment with AZD3965 highlighting the main detectable metabolites. **c** AZD3965-treated (but not vehicle-treated) tumours display decreased choline/water content following a 5-day treatment schedule. **d** AZD3965, but not vehicle, treatment reduces taurine/water levels in Raji tumours as measured by ^1^H MRS. **e** Tumour growth measurements show that while vehicle-treated Raji tumours grow larger over the 5 day experiment, AZD3965 treatment causes tumour stasis. **f** Ex vivo ^1^H NMR analysis of Raji tumour metabolites shows that AZD3965 causes increased tumour lactate confirming MCT1 blockade, concomitant with a reduction in PCho content. *P < 0.05; **P < 0.01; NS, not significant.

Tumour-bearing mice were anaesthetised with a mixture of air:oxygen:2% isoflurane and placed at the isocentre of a horizontal Bruker 7 Tesla microimaging system (Bruker Instruments, Ettlingen, Germany) with the tumours positioned in the centre of a 15 cm ^1^H surface coil. After shimming (linewidth on the unsuppressed water peak of ~15 Hz on average), localised ^1^H point resolved spectroscopy measurements of the tumours were performed prior to (day 1) and 2 h following the last dose (on day 5), as depicted in Fig. 1a, by acquiring water suppressed as well as unsuppressed spectra. Data were processed using jMRUI and AMARES fitting to determine chemical shift positions and peak integrals as previously described.^19^ After the last scan, the experiment was terminated with a schedule 1 method (cervical dislocation) and then tumours were excised, immediately snap frozen in liquid nitrogen and finally stored at −80 °C until further processing.

### In vitro NMR spectroscopy of cell and tumour extracts

Cells and xenograft tumour tissue were extracted as previously described^20^ and freeze-dried samples reconstituted in D_2_O containing 3-(trimethylsilyl) propionic-2,2,3,3-*d*_4_ acid (TSP) as an internal ^1^H NMR quantitation and chemical shift reference. Data were acquired on a Bruker 500 MHz spectrometer at 25 °C and processed as previously described.^16^ Metabolite content was measured by peak integration relative to TSP and normalised to cell number. For tumour metabolite analysis, peak areas of the metabolites of interest were normalised to tissue wet weight and expressed as arbitrary units/mg.

### Immune profiling of tumours

Fresh tumour tissue was collected and processed as previously described.^21^ Briefly, tumour fragments were digested in a mixture of collagenase and DNAse and disaggregated cell suspensions were treated with a red blood cell lysis buffer followed by cell number counting using trypan blue exclusion. To determine the frequency and phenotype of immune cells within tumours, multi-colour flow cytometry analysis of cell surface marker expression was applied using ~10^6^ live single cells and a standard protocol as previously described.^21^ Details of reagents used in tumour tissue digestion as well as flow cytometry, antibodies and gating strategy are provided in Supplementary Materials and methods and Supplementary Fig. S1.

### Isolation and processing of human peripheral blood mononuclear cells

Fresh blood samples (30 ml each) were donated by consented healthy volunteers (Ethics protocol number EudraCT 2010-024463-41), in accordance with the Declaration of Helsinki. Samples were collected in EDTA tubes and used to isolate peripheral blood mononuclear cells (PBMCs) using Ficoll-Hypaque density gradient centrifugation. Isolated PBMCs (2–15 million cells/sample) were incubated ex vivo in 0.1% DMSO or 25 nM AZD3965 for 1 h (in 10% fetal bovine serum (FBS) RPMI medium) and then harvested for hyperpolarised (HP) ^13^C NMR, a method that allows NMR signal enhancement by up to 10,000-fold, thus enabling the rapid and dynamic monitoring of metabolic reactions occurring within seconds.^22^ To monitor HP ^13^C-pyruvate-lactate exchange, PBMCs were transferred to a 5 mm NMR tube, incubated at 37 °C in FBS-free medium and ^13^C NMR spectra acquired with 2 s intervals and a 10° pulse for 4 min immediately after the addition of 10 mM HP [1-^13^C]pyruvic acid and 10 mM unlabelled lactate at pH 7 (total volume = 0.5 ml). Dynamic HP ^13^C time-series spectra were processed and the ratio of the areas under the curve for the lactate and pyruvate peaks (lactate_AUC_/pyruvate_AUC_) determined as previously described.^13^

### Statistical analysis

Single comparison two-tailed unpaired *t* test (for in vitro comparisons) and paired *t* test (for in vivo tumour changes prior to and following treatment) were used with *P* <0.05 considered statistically significant. Data represent the mean ± SE.

## Results

### MCT1 inhibition with AZD3965 decreases in vivo tumour choline phospholipid metabolism

To evaluate the impact of AZD3965 on tumour choline metabolism in vivo, we used non-invasive ^1^H MRS of Raji tumours treated with either vehicle or AZD3965 as depicted in Fig. 1a.

MRS is a clinically translatable technique for evaluating tumour metabolite profiles, with ^1^H MRS being the most commonly used method in the clinic, enabling the detection of metabolic species such as choline-related metabolites, taurine, creatine and lipids.^23^

Transverse anatomical images of a representative Raji tumour pre- and post-AZD3965 treatment together with corresponding in vivo ^1^H MR spectra are shown in Fig. 1b where the most prominent signals observed were from total choline (tCho), taurine and lipids. As shown in Fig. 1c, the tCho/water ratio decreased significantly in the AZD3965-treated tumours (81 ± 5% of pre-treatment values: *P* = 0.037), but remained unchanged in the vehicle-treated group (105 ± 14% of pre-treatment values: *P* = 0.57). Interestingly, the taurine/water ratio was also significantly reduced in the AZD3965-treated group (58 ± 6% compared to pre-treatment values, *P* = 0.02) as shown in Fig. 1d. The AZD3965-associated decrease in tCho/water was however independent of the fall in taurine since the reported tCho/water measurements were processed to exclude contributions from the overlapping taurine peak at the 3.2 p.p.m. position.

Tumour size measurements over the 5-day course of the experiment indicated that while the vehicle-treated tumours grew up to 1.4-fold relative to pre-treatment volumes (*P* = 0.01), the AZD3965-treated tumours showed cytostasis (*P* = 0.25), consistent with drug-induced tumour growth inhibition (Fig. 1e).

In vitro high-resolution ^1^H NMR analyses of excised Raji tumour extracts showed increased tumour lactate levels up to 1.65-fold in AZD3965-treated relative to vehicle-treated samples, thus confirming MCT1 blockade following AZD3965 treatment. Concomitant with this effect, we also observed decreased PCho levels in the drug-treated relative to control tumours (Fig. 1f).

These data indicate that treatment with AZD3965, at a dose and schedule that triggers MCT1 blockade in MCT4— Raji xenografts, causes a fall in tCho/water, detectable non-invasively using in vivo ^1^H MRS, as a result of decreased tumour PCho concentrations. This effect occurred prior to any drug-induced changes in tumour volume.

### AZD3965 reduces choline metabolism following lactate build-up

To delineate the basis for the fall in PCho and its relationship with the AZD3965-induced accumulation in tumour lactate, we used in vitro Raji cell cultures.

**Fig. 2:**
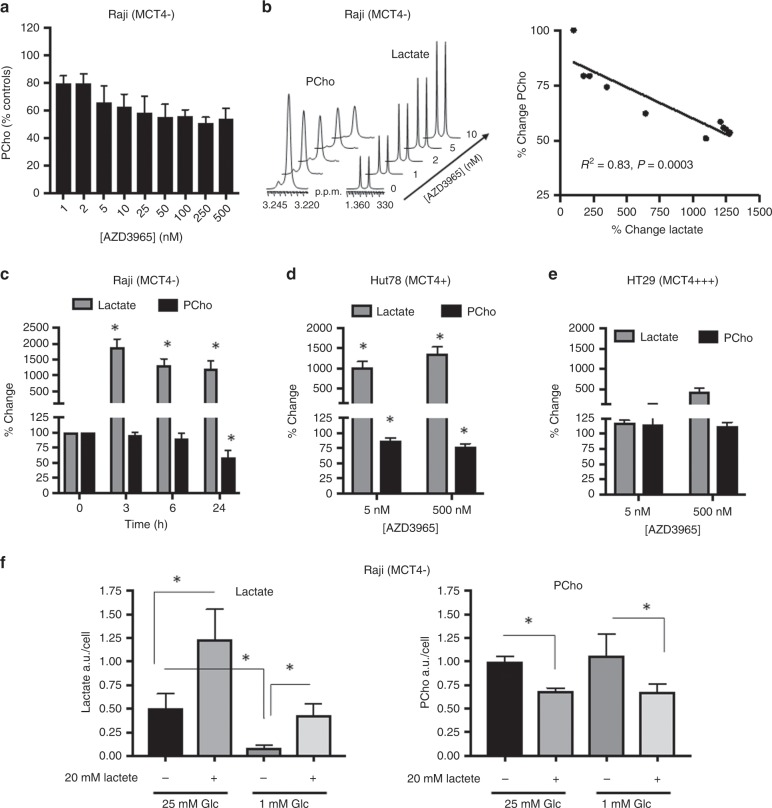
MCT1 inhibition with AZD3965 treatment decreases cellular PCho in Raji human lymphoma cells. **a** Concentration-dependent effects of AZD3965 on cellular PCho in Raji cells. **b** The concentration-dependent increase in cellular lactate following AZD3965 exposure correlates negatively with the reduction in cellular PCho. **c** AZD3965 (25 nM)-induced changes in Raji cellular PCho and lactate as a function of exposure time. **d** MCT4+ Hut78, but not MCT4+++ HT29 cells (**e**), display decreased PCho following exposure to AZD3965. **f** The effect of exogenous lactate supplementation and low glucose conditions on intracellular lactate and PCho levels in Raji cells. *P < 0.05.

^1^H NMR analysis indicated that the decrease in PCho triggered by AZD3965 exposure was concentration-dependent (Fig. 2a) and strongly negatively correlated with the extent of build-up in cellular lactate following a 24 h exposure to drug (Fig. 2b). The decline in PCho was not observed at early treatment time points (3 h, 6 h), indicating that it has slower dynamics relative to cellular lactate build-up (Fig. 2c) and implying that it may be related to downstream effects of treatment on gene/protein expression. We also observed a reduction in PCho in MCT4+ Hut78 human lymphoma cells following 24 h exposure to AZD3965, concomitant with substantial increases (up to 10-fold) in lactate_i_ (Fig. 2d) but not in MCT4+++ HT29 cells where lactate build-up was more modest following exposure to AZD3965 (Fig. 2e).

To examine whether the decrease in PCho was a result of lactate accumulation itself, rather than other drug-induced metabolic alterations, we cultured Raji cells in media supplemented with high levels of exogenous l-lactate (20 mM, pH ~7), to activate lactate uptake and force an increase in lactate_i_ (since lactate transport follows a concentration gradient^3^). The data showed that increasing lactate_i_ content under standard or low glucose conditions was sufficient to trigger a decrease in cellular PCho (Fig. 2f). In contrast, decreasing cellular lactate (by growing cells in low glucose conditions) did not impact PCho levels.

These findings show that the AZD3965-induced fall in cancer cell PCho levels is a downstream response to lactate_i_ accumulation, being more pronounced in cells with the greatest degree of increase in lactate following MCT1 blockade.

### AZD3965 inhibits phospholipid biosynthesis

**Fig. 3:**
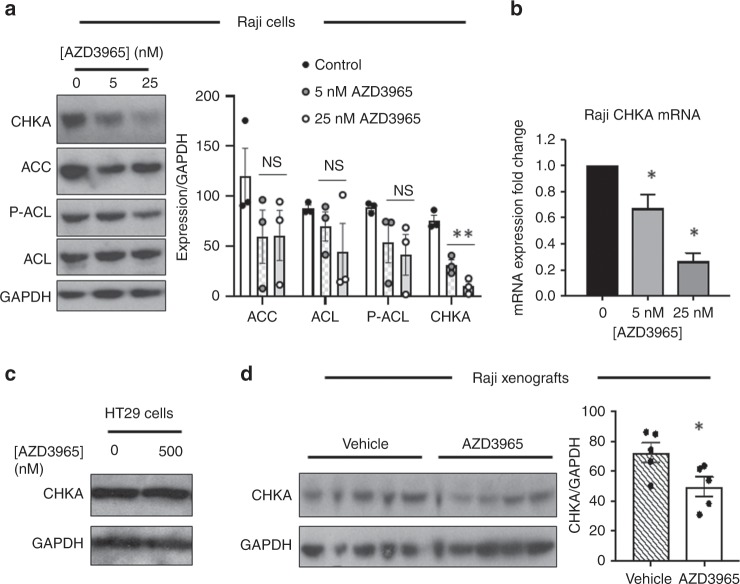
AZD3965 depletes CHKA levels. **a** Western blots and densitometric quantitation of bands in Raji human lymphoma cells showing depletion of CHKA following 24 h exposure to AZD3965 at concentrations that led to a fall in PCho. **b** RT-PCR analysis shows that AZD3965 (24 h treatment) decreases *CHKA* mRNA expression in Raji cells in a concentration-dependent manner. **c** CHKA protein levels are not changed in HT29 cells following 24 h exposure to AZD3965 as shown by western blot analysis. **d** Analysis of tumour tissue by western blotting confirms decreased CHKA protein in Raji tumours from mice treated with AZD3965 compared to vehicle-treated mice. Left panel shows CHKA band density quantitation. **P < 0.01; *P < 0.05; NS *P* ≥ 0.14.

PCho is produced through de novo synthesis from its precursor choline via choline kinase α (CHKA) as part of the Kennedy pathway for the synthesis of phosphatidylcholine, the most abundant cell membrane phospholipid. PCho can also be formed through release from phospholipid breakdown via phospholipases.^24^ Western blotting showed a concentration-dependent decrease in CHKA protein expression following 24 h exposure to AZD3965 in Raji cells (Fig. 3a). We also observed a trend for decreased expression of acetyl-CoA carboxylase (ACC) and phosphorylated (active) ATP citrate lyase, P-ACL, which are key enzymes in lipid biosynthesis^25,26^ (Fig. 3a), although this was not statistically significant (*P* ≥ 0.14).

qRT-PCR analysis of Raji cell *CHKA* messenger RNA (mRNA) expression showed significant decreases following exposure to AZD3965 (Fig. 3b), indicating that the fall in CHKA protein levels is driven by a reduction in its gene expression. No changes in CHKA protein expression were recorded in HT29 cells, in line with the lack of effect on intracellular PCho following AZD3965 exposure in these cells (Fig. 3c). Decreased CHKA protein expression was also confirmed by Western blot analysis in Raji tumour tissue obtained from AZD3965-treated mice (Fig. 3d), in concordance with the decline in tumour PCho content following drug treatment (as shown in Fig. 1f).

These data indicate that AZD3965 reduces PCho levels by inhibiting the expression of CHKA and de novo PCho formation, consistent with reduced lipogenesis.

### MCT1 blockade increases Raji tumour immune cell infiltration

To assess the cellular changes in the microenvironment of Raji tumours following disruption of lactate homeostasis, we used flow cytometry to determine the frequency and activation profile of tumour-infiltrating immune cells.

**Fig. 4:**
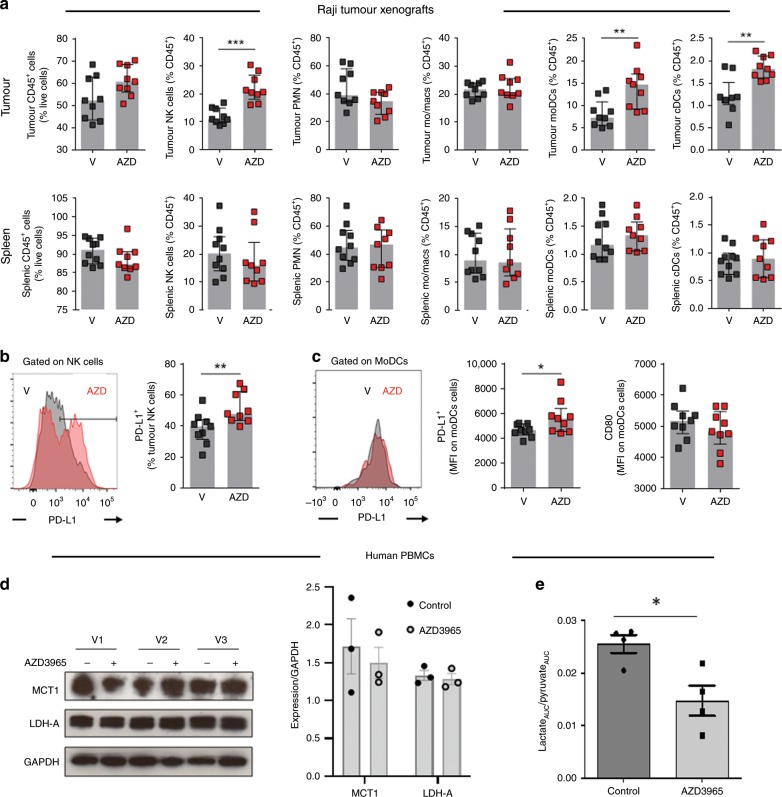
MCT1 blockade with AZD3965 modulates Raji tumour immune cell infiltration and MCT1 activity in human immune cells. **a** The frequency of leucocytes (CD45^+^), natural killer (NK) cells, neutrophils (PMNs), monocytes/macrophages (mo/macs), monocyte-derived DCs (moDCs) and conventional DCs (cDCs) in the tumour and spleen of Raji tumour-bearing mice treated with vehicle (V) or AZD3965 (AZD). **b** The percentage of tumour NK cells that were PD-L1^+^ and **c** the median fluorescence intensity (MFI) of PD-L1 and CD80 on tumour moDCs were all determined by flow cytometry. Individual mice along with median and interquartile range are depicted and the data represent *n* = 9 mice per group from two independent experiments. **d** Western blots with band quantitation (right) confirm the expression of MCT1 in human PBMCs in control and AZD3965-treated samples from three healthy volunteers (V1, V2, V3). **e** AZD3965 decreases HP ^13^C-lactate formation from ^13^C-pyruvate in human PBMCs consistent with the blockade of MCT1 in these cells. *P < 0.05; **P < 0.01; ***P < 0.001.

As shown in Fig. 4a (top panel), AZD3965-treated tumours showed increased abundance of both monocyte-derived and conventional dendritic cells (DCs) and natural killer (NK) cells, which are cells critical for antigen presentation and direct tumour cell killing, respectively. The frequency of monocytes, macrophages and neutrophils in the tumours was, in contrast, unaffected by AZD3965 treatment ((Fig. 4a, top panel), nor were the frequencies of immune cells in the periphery as indicated by spleen profiles (Fig. 4a, lower panel). Functional profiling indicated that there was an increase in mature NK cells in the tumour following AZD3965 treatment, as indicated by an increased proportion of PD-L1^+^ NK cells (Fig. 4b). Likewise, tumour-infiltrating DCs from AZD3965-treated mice had increased expression of PD-L1, but not CD80, suggesting an increased regulatory phenotype (Fig. 4b).

Although the changes in tumour immune cell infiltration and phenotype are likely to be a consequence of reduce lactate in the tumour microenvironment following MCT1 blockade,^15^ we wanted to assess the impact of AZD3965 treatment on MCT1 activity in immune cells themselves. For this we exposed freshly isolated human PBMCs (which include T, B and NK cells, monocytes and DCs^27^) to AZD3965 ex vivo and monitored HP ^13^C-lactate formation following the addition of HP ^13^C-pyruvate. Our results show that a 1 h treatment with 25 nM AZD3965 (a concentration previously shown to cause MCT1 inhibition in Raji cells) in human PBMCs (confirmed to express MCT1 and LDHA (Fig. 4d)) led to a fall in HP ^13^C-lactate levels as a result of decreased MCT1-mediated HP ^13^C-pyruvate uptake (Fig. 4e). These data are consistent with AZD3965 exerting direct effects on MCT1 activity (but not protein expression) in human PBMCs.

Taken together, these results indicate that AZD3965 modulates the tumour immune cell infiltrate in vivo, specifically increasing NK and DC abundance and maturation within the tumour, but not the periphery, despite exerting an inhibitory effect on MCT1 activity in immune cells.

## Discussion

Understanding the impact of MCT1 inhibition on overall tumour function together with identifying PD biomarkers for MCT1 modulation are key requirements for advancing the development of MCT1 inhibitors, which are in early phase clinical testing.

In this study, we show that at a treatment dose and schedule that led to tumour stasis, AZD3965 caused marked effects on tumour metabolic and immune phenotypes in a B cell lymphoma xenograft model representative of the clinical trial expansion patient cohort.

MCT1 inhibition with AZD3965 reduced Raji tumour choline content, as shown by ^1^H MRS of Cho/water, as a result of decreased tumour PCho levels. This effect occurred prior to any measured changes in tumour volume, indicating that ^1^H MRS of Cho/water has the potential as an early, non-invasive PD biomarker of MCT1 blockade.

The fall in PCho was recapitulated in vitro, showing a strong negative correlation with the extent of lactate_i_ build-up (being absent in the cell line with negligible lactate accumulation). Forced increase of cellular lactate was also sufficient to trigger a decrease in PCho, independently of AZD3965 treatment, indicating that the fall in PCho is a downstream cellular response to lactate_i_ build-up.

PCho can be synthesised from the phosphorylation of its precursor choline via CHKA, or it can be released following phospholipid breakdown via phospholipases.^24^ Our results indicate that the AZD3965-induced decrease in cellular PCho is likely to be due to a decline in its de novo synthesis from choline via reduced mRNA and protein expression of CHKA, which was also confirmed in Raji tumour tissue. Interestingly, AZD3965 treatment showed a trend towards inhibition of other rate-limiting lipogenic enzymes (ACC and P-ACL) in MCT4− Raji cells, potentially suggesting an overall decline in lipid biosynthesis following MCT1 inhibition in this model.

The inhibition of lipid biosynthesis observed with AZD3965 in the Raji model may in fact contribute to the anti-proliferative effects of the drug observed in our study given the role of lipogenesis in promoting cancer cell growth and proliferation.^25,26^ However, the mechanism underlying the link between lactate accumulation and decline in lipid biosynthetic enzyme expression, particularly *CHKA*, is unclear. Lactate has been shown to mediate gene expression regulation, including the activation of wound healing pathway genes and downregulation of lipid metabolism-related genes,^28,29^ with changes in cellular redox state invoked as one underlying mechanism.^30^ Whether similar or alternative mechanisms are at play here requires further investigation, particularly as previous reports have shown changes in redox state following MCT1 inhibitor treatment.^9^

Our in vivo investigation identifies new pathways that are modulated in the tumour following treatment with the MCT1 AZD3965 and shows the potential of non-invasive metabolic studies to provide biomarkers for reporting on accompanying downstream metabolic consequences of MCT1 inhibition. Non-invasive metabolic studies are a powerful tool for longitudinal assessment of metabolism at the tumour site, with methods such MRS (single voxel and spectroscopic imaging methods) enabling the steady state as well as dynamic assessment of several key metabolic pathways in pre-clinical models and patients.^31,32^ If validated in other MCT1-dependent models, our data support exploring the use of the clinically applicable ^1^H NMR spectroscopy of choline/water as a biomarker for monitoring the action of AZD3965 and potentially other MCT1 inhibitors in patient tumours.

One of the aims of our study was to assess the effects of AZD3965-induced alteration in metabolism on tumour biological phenotype. Our results show that MCT1 inhibition with AZD3965 increases tumour immune cell infiltration, involving NK cells and DCs, in the responsive MCT4− Raji human lymphoma xenograft model. NK cells exert direct cytolytic action on tumour cells and are a key component of the innate anti-tumour defence system while DCs enable antigen presentation to T cells and form a key part of adaptive anti-tumour immunity.^33^ Thus, increased tumour abundance of DC and NK cells could contribute to the growth inhibitory effects of AZD3965. A limitation of our findings is the fact that they were obtained in an immunodeficient mouse model (SCID mice lacking T and B cells), primarily due to current unavailability of suitable mouse models of MCT4−lymphoma. Nevertheless, our findings suggest a role for AZD3965 in stimulating anti-tumour immune responses involving both the innate and adaptive arms, which are likely to be involved in tumour growth inhibition following MCT1 blockade and which may be even more profound if reproduced in an immunocompetent setting.

Tumour microenvironment conditioning through reduced lactate export is likely to be the main mechanism for the increased tumour immune cell infiltration given the known inhibitory effects of lactate on anti-tumour immune cell activity and expansion, and the lack of effect on spleen immune cell phenotype.^34–36^ Interestingly, our human PBMC data also show that AZD3965 inhibits MCT1 in immune cells themselves potentially modulating their metabolism and also their activity.^37^ In agreement with this observation, previous work has shown that MCT1 inhibition has an immunosuppressive effect on T cells, preventing their expansion upon activation.^38^

Our data provide important new insights into the processes taking place in the tumour microenvironment, suggesting that the AZD3965-induced metabolic changes (particularly the fall in lactate export) are able to promote local anti-tumour immune cell expansion. Further studies in an immunocompetent setting are required to examine the effects of MCT1 inhibition on the function and phenotype of different immune cell subtypes involved in anti-tumour immunity including T cells.

It is noteworthy that AZD3965 treatment in our model also led to the upregulation of the immune checkpoint molecule PD-L1 on NK cells, previously shown to have a regulatory function in NK cells with regards to DC maturation and activation.^39^ Our data provide preliminary evidence for investigating the impact of AZD3965 on overall immune profiles and the potential for combination therapy with immune-modulating agents.

In summary, findings from our study provide valuable new insights into the pathways and mechanisms modulated in the tumour following MCT1 blockade, which could contribute to the anti-tumour activity of AZD3965. This knowledge may be exploited for furthering the development of AZD3965 and potentially other MCT1-targeted agents through the evaluation of non-invasive, clinically applicable response biomarkers, and exploring rational combination treatment to increase drug efficacy.

## Supplementary information

Supplementary data

## Data Availability

The datasets generated and analysed during the current study are available from the corresponding authors on reasonable request.
