# Supplementary material for: Monocarboxylate transporter 1 blockade with AZD3965 inhibits lipid biosynthesis and increases tumour immune cell infiltration
Source: Br J Cancer. 2020 Jan 15;122(6):895–903. doi: 10.1038/s41416-019-0717-x (PMC7078321; doi:10.1038/s41416-019-0717-x)
Supplement: Supplementary file 1 — Supplementary data [file 41416_2019_717_MOESM1_ESM.pdf]

Monocarboxylate transporter 1 blockade with AZD3965 inhibits lipid biosynthesis and increases tumour immune cell infiltration

Mounia Beloueche-Babari, Teresa Casals Galobart, Teresa Delgado-Goni, Slawomir Wantuch, Harold G Parkes, Debbie Tandy, James A Harker & Martin O Leach

1. Supplementary Materials and Methods

Antibodies for western blotting: The primary antibodies used for western blotting were mouse anti- glyceraldehyde-3-phosphate dehydrogenase (GAPDH) (Millipore, Nottingham, UK), rabbit anti- acetyl-CoA carboxylase, rabbit anti- ATP-citrate Lyase (ACL) and rabbit anti- phospho- ATP-citrate Lyase (P-ACL) (Cell Signaling Technology; Danvers, MA, USA). The secondary antibodies used were horseradish peroxidase linked anti-rabbit (GE Healthcare Life Sciences; Buckinghamshire, UK) and anti-mouse (Dako A/S, Glostrup, Denmark).

qRT-PCR analysis: Total cell RNA was extracted and qRT-PCR carried out as previously described [1]. The choline kinase α gene (*CHKA*)-specific primer and FAM labelled probe Hs00957875_m1 was used multiplexed with β-ACTIN-gene specific primers and VIC/TAMARA-labelled probe (Applied Biosystems). mRNA levels were determined for each sample in the same well relative to those of β-ACTIN (endogenous assay control) on the ABI 7900HT system and changes in *CHKA* mRNA levels expressed as the differences in the threshold cycle (2-ΔΔCt), inversely proportional to the amount of mRNA per sample.

Tumour processing and flow cytometry

Tumour tissue was finely chopped and incubated in RPMI containing 0.1 mg/ml collagenase D (Roche Diagnostics) and 25 µg/ml Dnase I (Roche Diagnostics) for 45 minutes at 37°C under gentle agitation. Single cell suspensions were generated by passing the tissue through

a 19G needle and then being passed through a 100 µm mesh cell strainer. Spleens were mashed through 100 µm cell strainers to generate a single cell suspension. Red blood cells were lysed using ACK buffer. Cells were then resuspended in PBS, counted by trypan blue. 2 x 10^6 cells per sample per panel were then stained with Zombie UV fixable viability dye (Biolegend) 1:1000 in PBS for 20 minutes at 4°C.

Surface antigens where then stained for using antibodies listed in the table below diluted in PBS containing 2% FBS and 2 mM EDTA for 30 minutes at 4°C and fixed using the Foxp3 fixation/permeabilization kit (eBiosience).

The cells were then incubated with antibodies against Ki67 and/or Granzyme B diluted in Perm buffer (eBioscience) for 1 h at 4°C. Cells were resuspended in PBS 2% FBS 2 mM EDTA and then run on a 5-laser BD Fortessa in the Flow Cytometry Core at South Kensington Campus of Imperial College London.

**Antibody**	**Clone**	**Company**	**Dilution**
**Myeloid panel**			
Brilliant violet (BV)-421 anti-mouse CD64	X54-5/7.1	Biolegend	1:100
BV510 anti-mouse Ly6G	1A8	Biolegend	1:200
BV605 anti-mouse CD11b	M1/70	Biolegend	1:200
BV711 anti-mouse PDL1	10F.9G2	Biolegend	1:200
FITC anti-mouse Ki67	16A8	eBioscience	1:100
PerCP-Cy5.5 anti-mouse IA/IE (MHCII)	55/114.15.2	Biolegend	1:200
PE anti-mouse CD80	16-10A1	eBioscience	1:100
PE-dazzle 594 anti-mouse CD11c	N418	Biolegend	1:200
Pe-Cy7 anti-mouse F4/80	BM8	Biolegend	1:200
APC anti-mouse CD45	30-F11	eBioscience	1:200
Alexa 700 anti-mouse CD19	6D5	Biolegend	1:50
**Innate lymphocyte panel**			
BV421 anti-mouse NKp46	29A1.4	Biolegend	1:100
BV605 anti-mouse KLRG1	2F1/KLRG1	Biolegend	1:100
BV711 anti-mouse PDL1	10F.9G2	Biolegend	1:200
FITC anti-mouse Ki67	16A8	Biolegend	1:100
PerCP Cy5.5 anti-mouse CD11b	M1/70	Biolegend	1:200
PE anti-human granzyme B	GB11	eBioscience	1:10
eFlour610 anti-mouse CD3e	145-2C11	eBioscience	1:300
PE-Cy7 anti-mouse PD1	RMP1-30	Biolegend	1:200
APC anti-mouse CD45	30-F11	eBioscience	1:200
Alexa 700 anti-mouse CD90.2	30-H12	Biolegend	1:100

Flow cytometry analysis

Flow cytometry was analyzed using FlowJo V10. Myeloid and lymphoid cells were gated as shown in figure S1.

2. Supplementary Figures

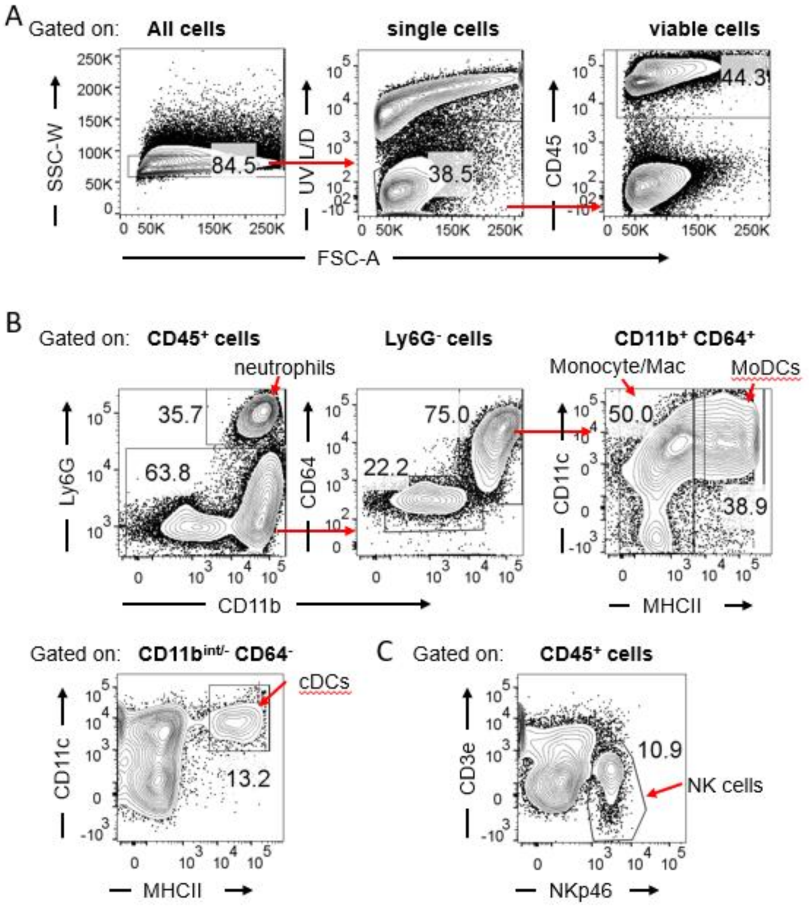

Supplementary figure S1. Gating strategy of immune cells by flow cytometry. Tumour and spleen tissue were analysed by flow cytometry. (A) single, viable, CD45+ cells were gated prior to further analysis. (B) After staining with a myeloid panel neutrophils (Ly6G$^+$ CD11b$^+$), monocyte/macrophages (CD11b$^+$CD64$^+$MHCII^{low}Ly6G$^-$), monocyte derived DCs (moDCs; CD11b$^+$CD64$^+$CD11c$^+$MHCII^{hi}Ly6G$^-$) and conventional DCs (cDCs; CD11c$^+$ MHCII^{hi} CD64^{low} CD11b$^{int/low}$) were gated as depicted. (C) Natural Killer (NK) cells were stained using a lymphocyte panel and gated as CD3e$^-$ NKp46$^+$. Data shown is tumour tissue from a vehicle control treated mouse.

References

1. Delgado-Goni T, Miniotis MF, Wantuch S, Parkes HG, Marais R, Workman P *et al.* The BRAF Inhibitor Vemurafenib Activates Mitochondrial Metabolism and Inhibits Hyperpolarized Pyruvate-Lactate Exchange in BRAF-Mutant Human Melanoma Cells. *Molecular cancer therapeutics* 2016; **15**(12): 2987-2999; doi 10.1158/1535-7163.MCT-16-0068.
